# The association of inflammasome and *TLR2* gene polymorphisms with susceptibility to tuberculosis in the Han Taiwanese population

**DOI:** 10.1038/s41598-020-67299-6

**Published:** 2020-06-23

**Authors:** Chi-Wei Liu, Chou-Jui Lin, Hui-Chun Hu, Hsiu-Jung Liu, Yu-Chi Chiu, Shih-Wei Lee, Lawrence Shih-Hsin Wu

**Affiliations:** 1 Taoyuan General Hospital, Ministry of Health and Welfare, Taoyuan, Taiwan; 2 Graduate Institute of Biomedical Sciences, China Medical University, Taichung, Taiwan

**Keywords:** Genetics, Medical research

## Abstract

Pulmonary tuberculosis (TB) caused by *Mycobacterium tuberculosis* (*Mtb*) is a global public health concern. Although inflammasome and the toll-like receptor 2 (*TLR2*) genes play an important role in host defense against *Mtb*, the associations of polymorphisms in these genes with TB risk are incompletely understood. A total of 230 TB patients and 213 individuals without TB were enrolled in this study. A significant difference in the frequencies of different *AIM2* rs2276405 genotypes between the non-TB and TB groups was detected. When the patients were stratified by gender or age, significant differences in genotype frequencies at *NLRP3* rs34298354 in men and in non-aged (≤65-year-old) subjects and at *IFI16* rs1772408 in women were found. OR analysis showed that the TC rs34298354 genotype in *NLRP3* was associated with reduced risk of TB. In women, the AG rs1772408 genotype in *IFI16* was associated with decreased TB risk. Haplotype analysis showed that, in comparison with the most common haplotype (T-T) of rs3804099-rs3804100 in the *TLR2* gene, the C-T haplotype was associated with an increased risk for TB. Our study indicates that rs34298354 in *NLRP3* and rs1772408 in *IFI16* protect individuals from TB, and that the less common *TLR2* haplotype is associated with increased TB susceptibility.

## Introduction

Tuberculosis (TB) is an infectious disease caused by *Mycobacterium tuberculosi*s (*Mtb*) that mostly affects the lungs. In their global tuberculosis report in 2019, the WHO estimated there were 10.0 million new TB cases and 1.2 million TB deaths among HIV-negative people in 2018^1^. In Taiwan, approximately 9759 new cases and 511 deaths occurred from mycobacterial infection in 2017^2^. About a quarter of the world’s population is infected with *Mtb* and thus at 5–10% lifetime risk of developing TB disease^1^. About 90% of people with a latent TB infection never develop active disease, suggesting that individual host factors (e.g., genetics, smoking, and alcohol) influence susceptibility to TB^3,4^. In recent years, significant relationships between genetic variation in host immune-related genes and TB risk have been reported^4–6^.

Inflammasomes are multiprotein complexes that form when cells sense invading infectious pathogens and that control antimicrobial host defenses^7,8^. The five major inflammasomes (NLRP1, NLRP3, NLRC4, Pyrin, and AIM2) include cytoplasmic and nuclear sensor molecules that form a complex with the effector protein pro-caspase-1^8^. The active caspase-1 processes pro-inflammatory interleukins (such as pro-IL-1β and IL-18) into their mature biologically active forms^9^. ESAT-6^10^, an *Mtb* protein, and transfected *Mtb* dsDNA^11,12^ can activate the NLRP3 and AIM2 inflammasomes, respectively. In addition, *Mtb* extracellular DNA can activate the IFI16 inflammasome, leading to the production of IFN-β^13^. AIM2-deficient mice show high susceptibility to *Mtb* due to impaired production of IL-18 and IFN-γ and reduced activation of caspase-1^14^. In recent years, associations of inflammasome gene polymorphisms with susceptibility to TB and the development of TB have been reported^4,15,16^. *NLRP3* polymorphism rs35829419 has been associated with extrapulmonary TB in Ethiopia^15^, and *IFI16* polymorphisms rs1101998 and rs1633256 have been associated with tuberculin skin test positivity in contacts of TB patients in Brazil^16^. However, few reports have evaluated the association of *AIM2* and *IFI16* gene polymorphisms with TB risk. Despite the importance of inflammasomes in the immune response to tuberculosis, it is still unknown whether inflammasome gene polymorphisms are associated with susceptibility to TB in the Han Taiwanese population.

Genetic polymorphism of toll‐like receptor 2 (*TLR2*), a TLR family member, influences the immune response to ESAT-6 in pulmonary tuberculosis patients^17^. TLRs are pattern recognition receptors expressed in antigen‐presenting cells and are important in host immunity to infectious pathogens^18,19^. They are involved in the recognition of *Mtb* and are linked to inflammasome activation^20^. The associations of gene polymorphisms in other TLR (*TLR1*, −4, −6, −8, −9, and −10) genes with TB risk have also been studied^21–23^. In Taiwan, genetic variants of *TLR2*, *TLR7*, and *TLR8* have been associated with increased risk for TB infection^24,25^. However, whether other *TLR2* polymorphisms are associated with genetic susceptibility to TB in the Han Taiwanese population is still unknown.

In light of the above information, we proposed that variants in inflammasome genes and *TLR2* could influence the host response to *Mtb* infection and the development of TB. In this study, we evaluated the association of inflammasome and *TLR2* gene polymorphisms with the susceptibility to TB. SNPs in inflammasome genes (*NLRP3*, *AIM2*, *IFI16*) and in *TLR2* were analyzed using TaqMan genotyping in subjects with and without TB. Our results indicate that genetic variants of inflammasome and *TLR2* genes are associated with TB risk in the Han Taiwanese population.

## Results

### Characteristics of the study subjects

443 adult subjects (including 213 patients with TB and 213 controls without TB infection) were enrolled in this study. In the Table 1, we found that TB patients had higher men/women ratio than controls, indicating men were more significant TB risk than women (*p* = 0.0001, χ^2^). TB subjects had a mean age of 57 years (range 20–91 years) and controls had a mean age of 66 years (range 20–97 years). Significant differences in age between the TB and non-TB groups were found in all subjects, men, and women by *t* test.

**Table 1:** The characteristics of the study participants.

Variables	Non-TB, N (%)	TB, N (%)	*p* value
Gender			
Man	124 (58.2)	173 (75.2)	0.0001^a^
Woman	89 (41.8)	57 (24.8)	
Age (years)			
Mean ± SD (range)	66 ± 19 (20~97)	57 ± 19 (20~91)	<0.0001^b^
Man, mean ± SD (range)	70 ± 17 (20~97)	59 ± 18 (20~91)	<0.0001^b^
Woman, mean ± SD (range)	61 ± 19 (23~94)	49 ± 20 (20~89)	0.008^b^
Age group-N (%)			
≤65	83 (39.0)	151 (65.7)	<0.0001^a^
>65	130 (61.0)	79 (34.3)	

SD = standard deviation; TB = tuberculosis; N = number of subjects; ^a^The statistical

analysis was tested by χ^2^-test; ^b^The statistical analysis was tested by *t*-test.

### Genotype distributions conformed to Hardy-Weinberg equilibrium

**Figure 1:**
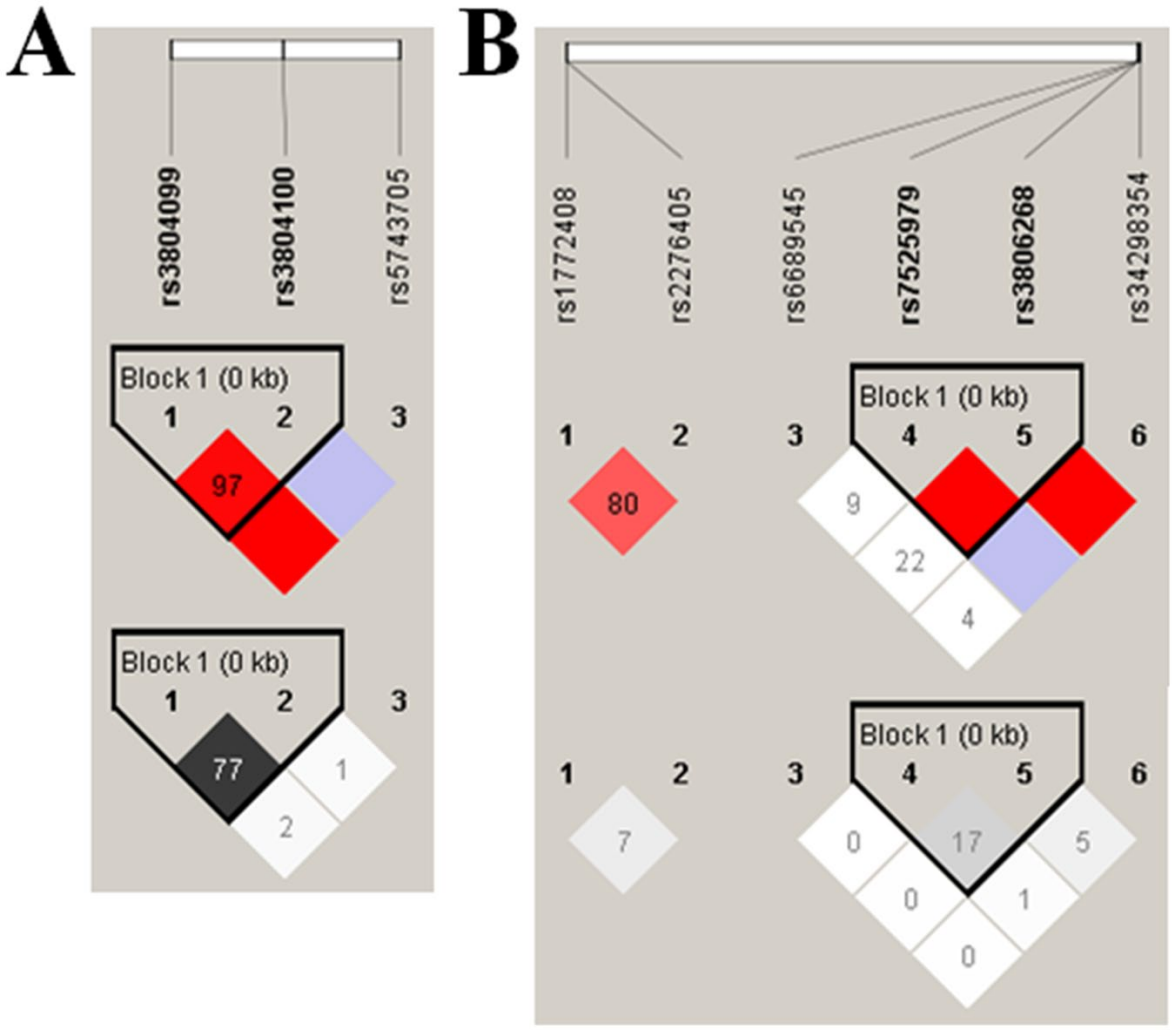
Linkage disequilibrium (LD) plot in D′ demonstrating adjacent strength between single nucleotide polymorphisms (SNPs) in the *AIM2*, *IFI16*, *TLR2*, and *NLRP3* genes. Patterns of LD between the *TLR2* SNPs in (**A**) and *NLRP3*, *AIM2*, and *IFI16* SNPs in (**B**). D′ and r^2^ values were multiplied by 100.

When the nine SNPs in the *AIM2*, *NLRP3*, *TLR2*, and *IFI16* regions were genotyped, none of their allelic distributions deviated from Hardy-Weinberg equilibrium (Table 2). The LD plot of the nine SNPs is shown in Fig. 1. One haploblock was identified at *NLRP3* and another at *TLR2*.

**Table 2:** Genotyping frequencies of SNPs in the TB and non-TB groups and results of logistic regression.

SNP ID	Location	Genotype	Genotype counts		*p* value^a^	Adj. odds ratio (95% CI)	*p* for OR^b^
			Non-TB (%)	TB (%)			
rs2276405	AIM2	TT	5 (2.3)	0 (0.0)	0.025		
(HW*p* = 0.274)	Exonic	TC	24 (11.3)	37 (16.1)		1.343 (0.747, 2.414)	0.324
	stop gain	CC (ref.)	184 (86.4)	193 (83.9)			
rs34298354	NLRP3	TT	0 (0.0)	0 (0.0)	0.079		
(HW*p* = 0.358)	Exonic	TC	32 (15.0)	22 (9.6)	(2*2 χ^2^ test)	0.536 (0.294, 0.979)	0.043
	synonymous	CC (ref.)	181 (85.0)	208 (90.4)			
rs3806268	NLRP3	GG	48 (22.5)	52 (22.6)	0.900	0.968 (0.556, 1.683)	0.907
(HW*p* = 0.580)	Exonic	AG	105 (49.3)	109 (47.4)		0.858 (0.539, 1.367)	0.520
	synonymous	AA (ref.)	60 (28.2)	69 (30.0)			
rs7525979	NLRP3	TT	2 (1.0)	5 (2.2)	0.570	2.119 (0.379, 11.841)	0.392
(HW*p* = 1.000)	Exonic	TC	48 (22.5)	53 (23.0)		1.025 (0.639, 1.643)	0.919
	synonymous	CC (ref.)	163 (76.5)	172 (74.8)			
rs6689545	NLRP3	CC	0 (0.0)	0 (0.0)	0.822		
(HW*p* = 1.000)	5′ near gene	TC	16 (7.5)	16 (7.0)	(2*2 χ^2^ test)	0.927 (0.426, 2.016)	0.848
		TT (ref.)	197 (92.5)	214 (93.0)			
rs3804099	TLR2	CC	15 (7.0)	24 (10.4)	0.384	1.529 (0.737, 3.171)	0.254
(HW*p* = 0.262)	Exonic	TC	79 (37.1)	88 (38.3)		1.138 (0.748, 1.733)	0.545
	Synonymous	TT (ref.)	119 (55.9)	118 (51.3)			
rs3804100	TLR2	CC	11 (5.2)	12 (5.2)	0.949	0.919 (0.372, 2.273)	0.855
(HW*p* = 0.789)	Exonic	TC	80 (37.5)	83 (36.1)		0.931 (0.614, 1.413)	0.737
	synonymous	TT (ref.)	122 (57.3)	135 (58.7)			
rs5743705	TLR2	CC	0 (0.0)	0 (0.0)	0.788		
(HW*p* = 0.650)	Exonic	TC	22 (10.3)	22 (9.6)	(2*2 χ^2^ test)	0.876 (0.454, 1.690)	0.692
	synonymous	TT (ref.)	191 (89.7)	208 (90.4)			
rs1772408	IFI16	AA	35 (16.4)	44 (19.1)	0.694	1.086 (0.603, 1.955)	0.783
(HW*p* = 0.541)	Intron variant	AG	112 (52.6)	113 (49.1)		0.871 (0.556, 1.365)	0.547
		GG (ref.)	66 (31.0)	73 (31.8)			

HWp: p value of Hardy-Weinberg disequilibrium test; ref: reference genotype; CI: confidence interval; OR: odds ratio. ^a^The statistical

analysis was tested by χ^2^-test; ^b^Adj. = adjusted for age and gender by logistic regression.

### Inflammasome and TLR2 gene polymorphisms associated with tuberculosis risk

Our results revealed a significant difference in *AIM2* SNP rs2276405 genotype frequencies between the non-TB and TB groups, whereas the other eight SNPs did not have significantly different genotype frequencies between TB patients and controls (Table 2). We used logistic regression to test the effect of interactions of age and genotype (divided into ≤65 and >65 years) and of gender and genotype, and found that the *p* values generated by logistic regression were all significant (Table 3). When the patients were stratified by gender, significant differences in the genotype frequencies of rs34298354 in men and rs1772408 in women were found (Table 4). When the patients were stratified by age, a significant difference in genotype frequencies of rs34298354 in non-aged (≤65-year-old) subjects was found (Table 5).

**Table 3:** Interaction of genetic variation and gender contribute to tuberculosis risk.

	χ^2^	df	*p* value
gender* genotype			
rs2276405	22.167	4^a^	<0.0001
rs34298354	19.257	3^b^	<0.0001
rs3806268	17.004	5	0.004
rs7525979	16.712	5	0.005
rs6689545	16.705	3^b^	0.001
rs3804099	18.653	5	0.002
rs3804100	15.271	5	0.009
rs5743705	15.009	3^b^	0.002
rs1772408	22.028	5	0.001
age*genotype			
rs2276405	40.480	5	<0.0001
rs34298354	36.157	3^b^	<0.0001
rs3806268	32.769	5	<0.0001
rs7525979	34.750	5	<0.0001
rs6689545	33.305	3^b^	<0.0001
rs3804099	33.294	5	<0.0001
rs3804100	33.205	5	<0.0001
rs5743705	32.012	3^b^	<0.0001
rs1772408	34.462	5	<0.0001

df: degree of freedom. ^a^rs2276405 TT genotype is only five subjects, none in male subjects and five in female subjects, so the df=4; ^b^rs34298354 TT genotype, rs6689545 CC genotype, and rs5743705 CC genotype are not detected in case and control groups, so the df=3. p value was calculated by logistic regression.

**Table 4:** Odds ratio analysis of AIM2 SNP (rs2276405), NLRP3 SNPs (rs34298354, rs3806268, rs7525979, rs6689545), TLR2 SNPs (rs3804099, rs3804100, rs5743705), and IFI16 SNP (rs1772408) in men and women with or without TB.

SNP ID	Genotype	Genotype counts		*p* value^a^	Adj. OR (95% CI)^b^	*p* for OR
		Non-TB	TB			
rs2276405						
Men	TT	0 (0.0)	0 (0.0)	0.136		
	TC	12 (9.7)	27 (15.6)	(2*2 χ^2^ test)	1.541 (0.728, 3.260)	*p* = 0.258
	CC (ref.)	112 (90.3)	146 (84.4)			
Women	TT	5 (5.6)	0 (0.0)	0.166		
	TC	12 (13.5)	10 (17.5)		1.071 (0.406, 2.822)	*p* = 0.890
	CC (ref.)	72 (80.9)	47 (82.5)			
rs34298354						
Men	TT	0 (0.0)	0 (0.0)	0.030		
	TC	23 (18.5)	17 (9.8)	(2*2 χ^2^ test)	0.540 (0.267, 1.090)	*p* = 0.085
	CC (ref.)	101 (81.5)	156 (90.2)			
Women	TT	0 (0.0)	0 (0.0)	0.788		
	TC	9 (10.1)	5 (8.8)	(2*2 χ^2^ test)	0.539 (0.162, 1.794)	*p* = 0.314
	CC (ref.)	80 (89.9)	52 (91.2)			
rs3806268						
Men	GG	31 (25.0)	37 (21.4)	0.742	0.826 (0.418, 1.631)	*p* = 0.582
	AG	61 (49.2)	87 (50.3)		0.916 (0.514, 1.631)	*p* = 0.766
	AA (ref.)	32 (25.8)	49 (28.3)			
Women	GG	17 (19.1)	15 (26.3)	0.393	1.356 (0.531, 3.461)	*p* = 0.524
	AG	44 (49.4)	22 (38.6)		0.727 (0.327, 1.617)	*p* = 0.434
	AA (ref.)	28 (31.5)	20 (35.1)			
rs7525979						
Men	TT	2 (1.6)	4 (2.3)	0.872	1.518 (0.249, 9.250)	*p* = 0.651
	TC	27 (21.8)	40 (23.1)		1.120 (0.626, 2.004)	*p* = 0.703
	CC (ref.)	95 (76.6)	129 (74.6)			
Women	TT	0 (0.0)	1 (1.8)	0.455		
	TC	21 (23.6)	13 (22.8)		0.872 (0.381, 1.994)	*p* = 0.746
	CC (ref.)	68 (76.4)	43 (75.4)			
rs6689545						
Men	CC	0 (0.0)	0 (0.0)	0.291		
	TC	12 (9.7)	11 (6.4)	(2*2 χ^2^ test)	0.587 (0.239, 1.440)	*p* = 0.245
	TT (ref.)	112 (90.3)	162 (93.6)			
Women	CC	0 (0.0)	0 (0.0)	0.294		
	TC	4 (4.5)	5 (8.8)	(2*2 χ^2^ test)	3.175 (0.717. 14.054)	*p* = 0.128
	TT (ref.)	85 (95.5)	52 (91.2)			
rs3804099						
Men	CC	9 (7.2)	15 (8.7)	0.584	1.257 (0.501, 3.156)	*p* = 0.626
	TC	43 (34.7)	68 (39.3)		1.234 (0.740, 2.060)	*p* = 0.420
	TT (ref.)	72 (58.1)	90 (52.0)			
Women	CC	6 (6.7)	9 (15.8)	0.210	2.053 (0.636, 6.624)	*p* = 0.229
	TC	36 (40.5)	20 (35.1)		0.954 (0.453, 2.011)	*p* = 0.902
	TT (ref.)	47 (52.8)	28 (49.1)			
rs3804100						
Men	CC	5 (4.0)	9 (5.2)	0.805	1.134 (0.351, 3.668)	*p* = 0.834
	TC	46 (37.1)	59 (34.1)		0.836 (0.502, 1.392)	*p* = 0.491
	TT (ref.)	73 (58.9)	105 (60.7)			
Women	CC	6 (6.7)	3 (5.3)	0.863	0.671 (0.145, 3.097)	*p* = 0.609
	TC	34 (38.2)	24 (42.1)		1.144 (0.557, 2.350)	*p* = 0.715
	TT (ref.)	49 (55.1)	30 (52.6)			
rs5743705						
Men	CC	0 (0.0)	0 (0.0)	0.766		
	TC	15 (12.1)	19 (11.0)	(2*2 χ^2^ test)	0.915 (0.434, 1.927)	*p* = 0.814
	TT (ref.)	109 (87.9)	154 (89.0)			
Women	CC	0 (0.0)	0 (0.0)	0.544		
	TC	7 (7.9)	3 (5.3)	(2*2 χ^2^ test)	0.735 (0.173, 3.121)	*p* = 0.676
	TT (ref.)	82 (92.1)	54 (94.7)			
rs1772408						
Men	AA	22 (17.7)	30 (17.3)	0.513	1.045 (0.518, 2.112)	*p* = 0.901
	AG	56 (45.2)	89 (51.5)		1.231 (0.719, 2.106)	*p* = 0.449
	GG (ref.)	46 (37.1)	54 (31.2)			
Women	AA	13 (14.6)	14 (24.6)	0.046	1.041 (0.371, 2.926)	*p* = 0.939
	AG	56 (62.9)	24 (42.1)		0.397 (0.173, 0.911)	*p* = 0.029
	GG (ref.)	20 (22.5)	19 (33.3)			

^*a*^*p* values were determined by the χ^2^ test. ^b^Adj. = adjusted for age by logistic regression; ref. = reference genotype.

**Table 5:** Odds ratio analysis of AIM2 SNP (rs2276405), NLRP3 SNPs (rs34298354, rs3806268, rs7525979, rs6689545), TLR2 SNPs (rs3804099, rs3804100, rs5743705), and IFI16 SNP (rs1772408) in non-aged (≤ 65-year-old) and aged (>65-year-old) subjects with or without TB.

SNP ID	Genotype	Genotype counts		*p* value^a^	Adj. OR (95% CI)^b^	*p* for OR
		Non-TB	TB			
rs2276405						
≤65	TT	2 (2.4)	0 (0.0)	0.134		
	TC	11 (13.3)	25 (16.6)		1.401 (0.632, 3.106)	*p* = 0.407
	CC (ref.)	70 (84.3)	126 (83.4)			
>65	TT	3 (2.3)	0 (0.0)	0.226		
	TC	13 (10.0)	12 (15.2)		1.573 (0.670, 3.689)	*p* = 0.298
	CC (ref.)	114 (87.7)	67 (84.8)			
rs34298354						
≤65	TT	0 (0.0)	0 (0.0)	0.038		
	TC	14 (16.9)	12 (7.9)	(2*2 χ^2^ test)	0.462 (0.196, 1.087)	*p* = 0.077
	CC (ref.)	69 (83.1)	139 (92.1)			
>65	TT	0 (0.0)	0 (0.0)	0.807		
	TC	18 (13.8)	10 (12.7)	(2*2 χ^2^ test)	0.720 (0.309, 1.680)	*p* = 0.447
	CC (ref.)	112 (86.2)	69 (87.3)			
rs3806268						
≤ 65	GG	14 (16.9)	32 (21.2)	0.727	1.274 (0.563, 2.882)	*p* = 0.561
	AG	42 (50.6)	72 (47.7)		0.898 (0.476, 1.691)	*p* = 0.738
	AA (ref.)	27 (32.5)	47 (31.1)			
>65	GG	34 (26.2)	20 (25.3)	0.926	0.855 (0.390, 1.875)	*p* = 0.696
	AG	63 (48.5)	37 (46.8)		0.847 (0.426, 1.684)	*p* = 0.636
	AA (ref.)	33 (25.3)	22 (27.9)			
rs7525979						
≤65	TT	1 (1.2)	3 (2.0)	0.716	1.370 (0.130, 14.396)	*p* = 0.793
	TC	21 (25.3)	32 (21.2)		0.818 (0.424, 1.577)	*p* = 0.549
	CC (ref.)	61 (73.5)	116 (76.8)			
>65	TT	1 (0.8)	2 (2.5)	0.340	2.953 (0.261, 33.461)	*p* = 0.382
	TC	27 (20.8)	21 (26.6)		1.406 (0.722, 2.739)	*p* = 0.317
	CC (ref.)	102 (78.4)	56 (70.9)			
rs6689545						
≤65	CC	0 (0.0)	0 (0.0)	0.337		
	TC	3 (3.6)	10 (6.6)	(2*2 χ^2^ test)	1.774 (0.456, 6.903)	*p* = 0.409
	TT (ref.)	80 (96.4)	141 (93.4)			
>65	CC	0 (0.0)	0 (0.0)	0.558		
	TC	13 (10.0)	6 (7.6)	(2*2 χ^2^ test)	0.733 (0.263. 2.042)	*p* = 0.553
	TT (ref.)	117 (90.0)	73 (92.4)			
rs3804099						
≤65	CC	7 (8.4)	19 (12.6)	0.574	1.975 (0.741, 5.263)	*p* = 0.173
	TC	30 (36.1)	56 (37.1)		1.105 (0.607, 2.011)	*p* = 0.743
	TT (ref.)	46 (55.5)	76 (50.3)			
>65	CC	8 (6.2)	5 (6.3)	0.913	1.010 (0.306, 3.332)	*p* = 0.987
	TC	49 (37.7)	32 (40.5)		1.206 (0.664, 2.192)	*p* = 0.539
	TT (ref.)	73 (56.1)	42 (53.2)			
rs3804100						
≤65	CC	5 (6.0)	10 (6.6)	0.968	1.186 (0.367, 3.838)	*p* = 0.775
	TC	30 (36.1)	56 (37.1)		1.127 (0.624, 2.035)	*p* = 0.691
	TT (ref.)	48 (57.9)	85 (56.3)			
>65	CC	6 (4.6)	2 (2.5)	0.566	0.539 (0.102, 2.847)	*p* = 0.467
	TC	50 (38.5)	27 (34.2)		0.823 (0.452, 1.498)	*p* = 0.524
	TT (ref.)	74 (56.9)	50 (63.3)			
rs5743705						
≤65	CC	0 (0.0)	0 (0.0)	0.844		
	TC	6 (7.2)	12 (7.9)	(2*2 χ^2^ test)	0.959 (0.333, 2.757)	*p* = 0.937
	TT (ref.)	77 (92.8)	139 (92.1)			
>65	CC	0 (0.0)	0 (0.0)	0.941		
	TC	16 (12.3)	10 (12.7)	(2*2 χ^2^ test)	0.966 (0.410, 2.275)	*p* = 0.937
	TT (ref.)	114 (87.7)	69 (87.3)			
rs1772408						
≤65	AA	12 (14.5)	31 (20.5)	0.322	1.275 (0.542, 2.995)	*p* = 0.578
	AG	47 (56.6)	71 (47.0)		0.786 (0.416, 1.482)	*p* = 0.456
	GG (ref.)	24 (28.9)	49 (32.5)			
>65	AA	23 (27.7)	13 (16.5)	0.906	1.111 (0.469, 2.633)	*p* = 0.812
	AG	65 (50.0)	42 (53.2)		1.218 (0.639, 2.323)	*p* = 0.549
	GG (ref.)	42 (32.3)	24 (30.3)			

^*a*^*p* values were determined by the χ^2^ test. ^b^Adj. = adjusted for gender by logistic regression; ref. = reference genotype.

The rs34298354 SNP in *NLRP3*, but none in the other three loci, was associated with susceptibility to TB. The TC heterozygous rs34298354 genotype was a reduced-risk genotype for susceptibility to TB, before and after adjusting for age and gender, compared with the CC genotype (aOR = 0.536; 95% CI = 0.294–0.979, *p* = 0.043; Table 2).

The rs1772408 SNP in *IFI16* was gender-dependent. In particular, the AG heterozygous rs1772408 genotype was associated with a reduced risk of TB in female subjects, adjusted for age (adjusted OR [aOR] = 0.397; 95% CI = 0.173–0.911, *p* = 0.029), compared with the AA genotype (Table 4). However, no significant association was found in non-aged (≤65-year-old) and aged (>65-year-old) subjects with or without TB group (Table 5).

### Haplotype and diplotype analyses

The results of LD analysis of the four loci in *NLRP3* and the three loci in *TLR2* are shown in Fig. 1. Of the four possible haplotypes in *NLRP3* rs7525979-rs3806268, three were detected in the non-TB and TB groups. In comparison with the most common haplotype, which includes only common alleles (C-A) of rs7525979-rs3806268, no haplotype was associated with a statistically significant increased risk of TB (Table 6). Four haplotypes of *TLR2* rs3804099-rs3804100 were detected in the non-TB and TB groups (Table 6). When we compared them with the most common haplotype, which includes only common alleles (T-T) of rs3804099-rs3804100, a significant association between the C-T haplotype and TB risk was found (aOR = 3.406; 95% CI = 1.546–7.505, *p* = 0.002). In addition, the difference in distribution of the *TLR2* haplotypes between the non-TB and TB groups was statistically significant (*p* = 0.014, χ^2^).

**Table 6:** Haplotype and diplotype distribution of the two investigated NLRP3 and TLR2 polymorphisms in control subjects and TB patients.

SNP		Frequency (%)	Non-TB (n)	TB (n)	*p* value^a^	Adj. OR (95% CI)^b^	*p* value for OR
rs7525979-rs3806268							
Haplotype	C-A (ref.)	53.3	225	247	0.678		
	C-G	33.7	149	150		0.937 (0.689, 1.274)	0.679
	T-G	13.0	52	63		1.083 (0.702, 1.670)	0.720
Diplotype	C-A/C-A (ref.)		60	69			
	C-G/any		124	121		0.865 (0.546, 1.369)	0.535
	T-G/any		50	58		0.969 (0.565, 1.662)	0.910
rs3804099-rs3804100							
Haplotype	T-T (ref.)	72.0	315	323	0.014		
	C-C	23.2	100	106		1.011 (0.724, 1.411)	0.949
	C-T	4.4	9	30		3.406 (1.546, 7.505)	0.002
	T-C	0.4	2	1		0.810 (0.066, 9.988)	0.870
Diplotype	T-T/T-T (ref.)		118	117			
	C-C/any		90	94		1.042 (0.691, 1.572)	0.843
	C-T/any		9	30		3.513 (1.550, 7.962)	0.003
	T-C/any		2	1		0.834 (0.067, 10.402)	0.888

*p values were determined by the χ^2^ test; ^b^Adj. = adjusted for age and gender by logistic regression; ref. = reference genotype; TB = tuberculosis; OR = odds ratio.

The association observed in the haplotype analyses was also found in the diplotype analyses (Table 6). In *NLRP3*, no significant association between any diplotype of *NLRP3* rs7525979-rs3806268 was found when compared with the homozygous C-A/C-A diplotype. In *TLR2*, we observed that carriers of at least one C-T haplotype of rs3804099-rs3804100 had increased TB risk in comparison with the individuals carrying the homozygous T-T/T-T diplotype (aOR = 3.513; 95% CI = 1.550–7.962, *p* = 0.003).

## Discussion

Previous studies have found associations of *NLRP3* and *TLR2* polymorphisms with susceptibility to TB^5,15–17,19,21^. In our study of the Taiwanese population, we found that the TC genotype of *NLRP3* rs34298354 was associated with decreased risk of TB. In addition, we found that the AG genotype of *IFI16* rs1772408 was gender-dependently associated with reduced risk for TB. In haplotype analysis, we found that the C-T haplotype of *TLR2* rs3804099-rs3804100 was associated with increased susceptibility to TB. This indicates that *NLRP3*, *IFI16*, and *TLR2* play an important role in protection against TB infection in the Han Taiwanese population.

We found no significant association between TB risk and rs3804099 and rs3804100 in *TLR2*, similar to the finding of no significant association of these SNPs with TB risk in a Western Chinese population^26^. However, they have been associated with TB risk in the Tibetan Chinese population^27^, suggesting a possible ethnicity-specific effect of rs3804099 and rs3804100 on susceptibility to TB. Using Haploview v.4.2 to evaluate linkage disequilibrium, one haploblock (rs3804099-rs3804100) in *TLR2* was found. This haploblock has been reported in multiple studies^26–29^. The rs3804099-rs3804100 haplotype has been reported to be associated with risks of tuberculosis^27^, hepatocellular carcinoma^28^, and allergic asthma^29^. In this study, haplotype analysis of two variations in *TLR2* (rs3804099 and rs3804100) showed significant association of the C-T haplotype with increased risk of TB (Table 6). A previous study found that peripheral blood leukocytes from trauma patients with the *TLR2* rs3804099 CC genotype produce greater amounts of IL-10, IL-8, and TNF-α than those having the TT genotype, after bacterial lipoprotein stimulation^30^. In another study, TB patients with the rs3804100 CC genotype had significantly higher blood absolute NK cell counts at diagnosis than those carrying the T allele^24^. Therefore, the C-T haplotype of *TLR2* rs3804099-rs3804100 may influence TB infection by affecting cytokine production or NK cell counts.

In this study, we found that *NLRP3* rs34298354 and the *TLR2* rs3804099-rs3804100 haplotype were associated with susceptibility to TB. However, rs34298354, rs3804099, and rs3804100 are exonic, synonymous SNPs. In the GTExPortal database, rs3804099 and rs3804100 are reported to be associated with *TLR2* expression (according to expression quantitative trait loci analysis) in whole blood and some tissues (https://gtexportal.org/home/snp/rs3804099; https://gtexportal.org/home/snp/ rs3804100). The synonymous genetic variants alter mRNA splicing, mRNA stability, mRNA structure, and protein folding^31^. In *MDR1*, the synonymous SNP C3435T is reported to alter protein activity/substrate specificity^32^. Peripheral blood mononuclear cells from subjects with the *NLRP3* rs34298354 CC genotype had higher IL-1β levels than those from subjects with the CT genotype after stimulation with dead mycobacterium avium complex (MAC) bacilli and lipopolysaccharide^33^. In this study, we did not investigate whether the associations of rs34298354, and rs3804099 and rs3804100, with TB risk were due to respective changes in *NLRP3* or *TLR2* gene expression or protein activity/substrate specificity, but this should be done in the future.

The TB statistics of the Taiwan CDC showed that TB prevalence in men is higher than in women, with a ratio of 2.2:1 in 2017^2^. Accordingly, we separated men from women to examine the possibility of a difference in TB prevalence between non-TB and TB subjects (Table 4). Our results indicate that the *IFI16* rs1772408 AG genotype is associated with a reduced risk of TB in women. However, no report has indicated that this genotype is related to regulation of *IFI16* gene expression and the development of disease. Similar to previous studies in the Han Taiwanese population, *SOCS3* SNPs rs4331426 in women and rs35037722 in men were associated with TB^6,34^ and *NLRP3* SNPs rs3806268 and rs34298354 in women and *TLR2* SNP rs3804100 in men were associated with MAC^33^. In addition, bisphenol A, an environmental estrogen, stimulates IFI16 protein expression in human peripheral blood mononuclear cells^35^, suggesting a possible gender-dependent association of *IFI16* gene polymorphism with TB risk. rs1772408 is located in the seventh intron of *IFI16*. Intronic polymorphisms can act as enhancers or silencers that regulate mRNA splicing^36^. In addition, the database GTExPortal reports a significant association between different *IFI16* rs1772408 genotypes and *IFI16* expression in skin (https://gtexportal.org/home/snp/rs1772408). Thus, rs1772408 may exert a gender-dependent effect on susceptibility to TB by regulating *IFI16* expression. A future study that involves a larger number of TB and non-TB subjects and compares *IFI16* expression levels among different genotypes will help solidify the association of rs1772408 with susceptibility to TB.

Our study has some limitations: First, the sample size was relatively small and some significance may have been under-estimated. According to the method of Levine *et al*.^37^ by using the Post-hoc Power Calculator on web (https://clincalc.com/stats/ Power.aspx), the statistical powers for ORs ratio analysis of rs34298354 TC genotype in total subjects, rs1772408 AG genotype in women, and rs3804099-rs3804100 C-T haplotype in total subjects were 0.41, 0.70, and 0.90, respectively. Except haplotype analysis, the total number of subjects provided low statistical power (<0.8) in this study. Thus, a future study to further increase the number of each grouped subjects will help solidify our finding. Second, the selected participants were enroll in Taiwan, which means our study was targeting mainly the susceptibility of TB in the Han Taiwanese population. Thus, our findings may not be generalized to other ethnicities and areas. Third, we did not collect the information about TB contact and latent TB infection diagnosis in our study. The influence of these factors on TB development has been suggested, and this may affect the assay results.

Our result showed a significant difference in *AIM2* rs2276405 genotype frequencies between subjects with and without TB. In addition, there were significant differences in the genotype frequencies of *NLRP3* rs34298354 in men and non-aged subjects and of *IFI16* rs1772408 in women. After the OR was adjusted for age and gender, AG heterozygotes at *NLRP3* rs34298354 showed reduced risk of TB, and AG heterozygosity at *IFI16* rs1772408 showed a gender-dependent influence on TB susceptibility. Through haplotype analysis, we found that the C-T haplotype at *TLR2* rs3804099-rs3804100 was associated with an increased risk for TB compared with the T-T haplotype. We conclude that *NLRP3*, *IFI16*, and *TLR2* polymorphisms are associated with TB risk in the Han Taiwanese population.

## Subjects and methods

### Study population

In this prospective study, all participants (297 men, 146 women) were recruited from General Taoyuan Hospital (Taoyuan, Taiwan) from January 2016 to December 2019. The inclusion criteria for TB group were as follows: adult patients (20 to 99 years old) diagnosed with active TB, with evident TB lesions on simple X-ray and computed tomography, and positive sputum smears and cultures for mycobacteria. For the control group, 213 adult volunteers with normal chest radiographs and without active TB or a history of TB were enrolled. All participants infected with human immunodeficiency virus (HIV) or treated with immunosuppressive drugs were excluded. According to the Rosner’s method^38^, the minimum sample size required for the experiment was performed by sample size calculator on web (https://clincalc.com/Stats/SampleSize.aspx). When anticipated incidence was set to detect the difference between two independent study groups (case and control) with a dichotomous primary endpoint showing 45% and 31% genotype frequencies in dominant model (odds ratio = 1.9), respectively, and reach to an α (type I error rate) of 0.05 and β of 0.2 (power=0.8), the minimum sample size needed for our study was 376 (188 for each group). An informed consent form was signed by each patient and volunteer enrolled in the study. The study protocol conformed to the ethical guidelines of the 1975 Declaration of Helsinki and was approved by the Medical Ethics and Institutional Review Board of the Taoyuan General Hospital, Ministry of Health and Welfare, Taoyuan, Taiwan.

### DNA purification from buccal swabs

According to the methods of Wu *et al*.^6^, genomic DNA was purified from oral swabs collected from the 443 subjects using a QIAamp DNA Mini Kit (Qiagen, Valencia, CA, USA). Briefly, the buccal swab placed in a 2-ml microcentrifuge tube with 400 µl PBS, 20 µl QIAGEN protease stock solution and 400 µl Buffer AL and then incubated at 56 °C for 10 min to lyse the cells. After mixing the cell lysate with 400 µl of absolute ethanol, the supernatant after centrifugation at 6,000 × *g* for 1 min was applied to a QIAamp Mini spin column for DNA purification and washed twice with buffer AW1 and AW2. DNA was eluted from the spin column with Buffer AE or sterile distilled deionized water (150 µl) for a 1-min incubation at room temperature before centrifugation at 6,000 ×*g* for 2 min. The purified DNA concentration was quantified by spectrophotometry at 260 nm and then stored at −80 °C until SNP genotyping by TaqKey Science Co., LTD (Hualien, Taiwan).

### SNP genotyping assays

We chose nine SNPs to study the association of inflammasome genes and *TLR2* with TB susceptibility. The tag SNPs of the *AIM2*, *NLRP3*, *IFI16*, and *TLR2* genomic regions were selected according to the SeattleSNPs website (https://gvs.gs.washington.edu/GVS150/)^39^. The SeattleSNPs database showed *AIM2* tag SNP rs2276405; *NLRP3* tag SNPs rs3806268, rs7525979, and rs6689545; *TLR2* tag SNPs rs3804099 and rs3804100; and *IFI16* tag SNP rs1772408 (all tag SNPs with minor allele frequency >0) used for the study in Han Chinese Beijing (HCB). In addition, rs34298354 in *NLRP3* and rs5743705 in *TLR2* were selected on the basis of a prior report^33^. All SNP genotyping was performed using TaqMan SNP Genotyping Assays^40^. The primers and probes for the selected SNPs were from an ABI assay on demand (AOD) kit (cat. #4351379, Thermo Fisher Scientific Inc., MA, USA). Reactions were carried out according to the manufacturer’s protocol (TaqMan SNP Genotyping Assays, protocol, Part Number 4332856 Rev. C). The probe fluorescence signal was detected using an ABI Prism 7900 Real-Time PCR System.

### Statistical analysis

All statistical analyses were performed using SPSS version 20.0 (IBM Corp., Armonk, NY, USA). Genotype deviations from Hardy-Weinberg equilibrium were assessed and the differences in genotype frequencies between the non-TB and TB groups were tested using the *χ*^2^ test^6^. Intermarker linkage disequilibrium (LD) measures r^2^ and D′ were estimated and haplotype blocks were defined using Haploview v.4.2^41^. The odds ratios (ORs) and 95% confidence intervals (CIs) were calculated from contingency tables^42^. The associations between different genotypes and TB were estimated by odds ratios (ORs) from univariate and multivariate logistic regression analyses adjusted by age and gender.

## References

[1] World Health Organization. Global Tuberculosis Report 2019. World Health Organization Genova, https://www.who.int/tb/publications/global_report/en/, (2019).

[2] Taiwan, R.O.C. Center for Disease Control, Department of Health. TB Statistics. https://daily.cdc.gov.tw/stoptb/CareMagChart.aspx, (2019).

[3] Marimani M, Ahmad A, Duse A (2018). The role of epigenetics, bacterial and host factors in progression of Mycobacterium tuberculosis infection. Tuberculosis (Edinb).

[4] Cai L (2019). The Research Progress of Host Genes and Tuberculosis Susceptibility. Oxid. Med. Cell Longev..

[5] Souza de Lima D, Ogusku MM, Sadahiro A, Pontillo A (2016). Inflammasome genetics contributes to the development and control of active pulmonary tuberculosis. Infect. Genet. Evol..

[6] Lin CJ (2019). Polymorphisms of suppressor of cytokine signaling-3 associated with susceptibility to tuberculosis among Han Taiwanese. Cytokine.

[7] Lupfer C, Malik A, Kanneganti TD (2015). Inflammasome control of viral infection. Curr. Opin. Virol..

[8] Broz P, Dixit VM (2016). Inflammasomes: mechanism of assembly, regulation and signalling. Nat. Rev. Immunol..

[9] Sharma D, Kanneganti TD (2016). The cell biology of inflammasomes: Mechanisms of inflammasome activation and regulation. J. Cell Biol..

[10] Mishra BB (2010). Mycobacterium tuberculosis protein ESAT-6 is a potent activator of the NLRP3/ASC inflammasome. Cell Microbiol..

[11] Shah S (2013). Cutting edge: Mycobacterium tuberculosis but not nonvirulent mycobacteria inhibits IFN-beta and AIM2 inflammasome-dependent IL-1beta production via its ESX-1 secretion system. J. Immunol..

[12] Ablasser A, Dorhoi A (2016). Inflammasome activation and Function during infection with *Mycobacterium tuberculosis*. Curr. Top. Microbiol. Immunol..

[13] Briken V, Ahlbrand SE, Shah S (2013). Mycobacterium tuberculosis and the host cell inflammasome: a complex relationship. Front. Cell Infect. Microbiol..

[14] Saiga H (2012). Critical role of AIM2 in Mycobacterium tuberculosis infection. Int. Immunol..

[15] Abate E (2019). Polymorphisms in CARD8 and NLRP3 are associated with extrapulmonary TB and poor clinical outcome in active TB in Ethiopia. Sci. Rep..

[16] Cubillos-Angulo JM (2019). Polymorphisms in interferon pathway genes and risk of *Mycobacterium tuberculosis* infection in contacts of tuberculosis cases in Brazil. Int. J. Infect. Dis..

[17] Mandala JP (2019). Toll-like receptor 2 polymorphisms and their effect on the immune response to ESAT-6, Pam3CSK4 TLR2 agonist in pulmonary tuberculosis patients and household contacts. Cytokine.

[18] Akira S, Uematsu S, Takeuchi O (2006). Pathogen recognition and innate immunity. Cell.

[19] Mukherjee S, Huda S, Sinha Babu SP (2019). Toll-like receptor polymorphism in host immune response to infectious diseases: A review. Scand. J. Immunol..

[20] Mortaz E (2015). Interaction of pattern recognition receptors with *Mycobacterium tuberculosis*. J. Clin. Immunol..

[21] Schurz H, Daya M, Möller M, Hoal EG, Salie M (2015). TLR1, 2, 4, 6 and 9 variants associated with tuberculosis susceptibility: A systematic review and meta-analysis. PLoS One.

[22] Salie M (2015). Association of toll-like receptors with susceptibility to tuberculosis suggests sex-specific effects of TLR8 polymorphisms. Infect. Genet. Evol..

[23] Wang Y (2018). Polymorphisms in toll-like receptor 10 and tuberculosis susceptibility: Evidence from three independent series. Front. Immunol..

[24] Chen YC (2010). Toll-like receptor 2 gene polymorphisms, pulmonary tuberculosis, and natural killer cell counts. BMC Med. Genet..

[25] Lai YF (2016). Functional polymorphisms of the TLR7 and TLR8 genes contribute to *Mycobacterium tuberculosis* infection. Tuberculosis (Edinb.).

[26] Zhang J (2018). Importance of common TLR2 genetic variants on clinical phenotypes and risk in tuberculosis disease in a western Chinese population. Infect. Genet. Evol..

[27] Xue X (2017). The association analysis of TLR2 and TLR4 gene with tuberculosis in the Tibetan Chinese population. Oncotarget.

[28] Junjie X (2012). The association between Toll-like receptor 2 single-nucleotide polymorphisms and hepatocellular carcinoma susceptibility. BMC Cancer.

[29] Bjørnvold M (2009). A TLR2 polymorphism is associated with type 1 diabetes and allergic asthma. Genes. Immun..

[30] Chen KH (2011). Identification of haplotype tag SNPs within the entire TLR2 gene and their clinical relevance in patients with major trauma. Shock.

[31] Hunt R, Sauna ZE, Ambudkar SV, Gottesman MM, Kimchi-Sarfaty C (2009). Silent (synonymous) SNPs: should we care about them?. Methods Mol Biol..

[32] Komar AA (2007). Silent SNPs: impact on gene function and phenotype. Pharmacogenomics.

[33] Wu MF (2019). NLRP3 inflammasome is attenuated in patients with Mycobacterium avium complex lung disease and correlated with decreased interleukin-1β response and host susceptibility. Sci. Rep..

[34] Lee SW (2016). SNP rs4331426 in 18q11.2 is associated with susceptibility to tuberculosis among female Han Taiwanese. J. Microbiol. Immunol. Infect..

[35] Panchanathan R, Liu H, Leung YK, Ho SM, Choubey D (2015). Bisphenol A (BPA) stimulates the interferon signaling and activates the inflammasome activity in myeloid cells. Mol. Cell Endocrinol..

[36] Cooper DN (2010). Functional intronic polymorphisms: Buried treasure awaiting discovery within our genes. Hum. Genomics.

[37] Levine M, Ensom MH (2001). Post hoc power analysis: an idea whose time has passed?. Pharmacotherapy.

[38] Rosner, B. Fundamentals of Biostatistics, 7th ed. (Boston: Brooks/Cole, Cengage Learning 2011).

[39] NHLBI Program for Genomic Applications. *SeattleSNPs*, https://gvs.gs.washington.edu/GVS150 (2019).

[40] Woodward J (2014). Bi-allelic SNP genotyping using the TaqMan assay. Methods Mol. Biol..

[41] Barrett JC, Fry B, Maller J, Daly MJ (2005). Haploview: analysis and visualization of LD and haplotype maps. Bioinformatics.

[42] Morris JA, Gardner MJ (1998). Calculating confidence intervals for relative risks (odds ratios) and standardised ratios and rates. Br. Med. J. (Clin Res Ed)..

